# A Comparative Study of the Plasma Chemokine Profile in COVID-19 Patients Infected with Different SARS-CoV-2 Variants

**DOI:** 10.3390/ijms23169058

**Published:** 2022-08-13

**Authors:** Zoia R. Korobova, Natalia A. Arsentieva, Natalia E. Liubimova, Vladimir G. Dedkov, Anna S. Gladkikh, Alena A. Sharova, Ekaterina I. Chernykh, Victor A. Kashchenko, Vyacheslav A. Ratnikov, Victor P. Gorelov, Oksana V. Stanevich, Alexandr N. Kulikov, Dmitriy E. Pevtsov, Areg A. Totolian

**Affiliations:** 1 Saint Petersburg Pasteur Institute, 14 Ulitsa Mira, 197101 St. Petersburg, Russia; 2 Intensive Care Unit, Department of Immunology, Department of Infectious Diseases, Pavlov First State Medical University of St. Petersburg, 6-8 Ulitsa L’va Tolstovo, 197022 St. Petersburg, Russia; 3 Clinical Hospital 122 of the North-Western Scientific and Clinical Center Named after L.G. Sokolov, Prospekt Kul’tury, 4, 194291 St. Petersburg, Russia; 4 Department of Faculty Surgery, Saint Petersburg State University, Universitetskaya Naberezhnaya, 7/9, 199034 St. Petersburg, Russia; 5 Research Institute of Influenza, Ulitsa Professora Popova, 16/17, 197022 St. Petersburg, Russia

**Keywords:** COVID-19, chemokines, macrophage-derived chemokine, SARS-CoV-2 variants, Omicron

## Abstract

Background. Infection caused by SARS-CoV-2 mostly affects the upper and lower respiratory tracts and causes symptoms ranging from the common cold to pneumonia with acute respiratory distress syndrome. Chemokines are deeply involved in the chemoattraction, proliferation, and activation of immune cells within inflammation. It is crucial to consider that mutations within the virion can potentially affect the clinical course of SARS-CoV-2 infection because disease severity and manifestation vary depending on the genetic variant. Our objective was to measure and assess the different concentrations of chemokines involved in COVID-19 caused by different variants of the virus. Methods. We used the blood plasma of patients infected with different variants of SARS-CoV-2, i.e., the ancestral Wuhan strain and the Alpha, Delta, and Omicron variants. We measured the concentrations of 11 chemokines in the samples: CCL2/MCP-1, CCL3/MIP-1α, CCL4/MIP-1β, CCL7/MCP-3, CCL11/Eotaxin, CCL22/MDC, CXCL1/GROα, CXCL8/IL-8, CXCL9/MIG, CXCL10/IP-10, and CX3CL1/Fractalkine. Results. We noted a statistically significant elevation in the concentrations of CCL2/MCP-1, CXCL8/IL-8, and CXCL1/IP-10 independently of the variant, and a drop in the CCL22/MDC concentrations. Conclusions. The chemokine concentrations varied significantly depending on the viral variant, leading us to infer that mutations in viral proteins play a role in the cellular and molecular mechanisms of immune responses.

## 1. Introduction

Since March 2020, the emergence of the COVID-19 pandemic has brought new challenges to global healthcare and science. Caused by a single-stranded RNA genome virus, this infection is known to cause a variety of symptoms ranging from the common cold to pneumonia with acute respiratory distress syndrome [1].

Infection caused by SARS-CoV-2 mostly affects the upper and lower respiratory tracts, particularly the lung tissue, due to the affinity of the spike protein of the virion towards the ACE2 receptor [2]. This receptor is extensively found in different types of body cells, including epithelia of the respiratory tract and the endothelium of the vascular system. In COVID-19 pathogenesis, there is also the factor of its involvement in lung fibrogenesis and protection against lung tissue damage. In both blocking the activation of the ACE2/MAS/G protein axis—a crucial part of vascular dilatation—and causing local inflammation, SARS-CoV-2 infection leads to lung tissue damage [3]. Inflammatory process in the respiratory tract eventually progresses to pneumonia and respiratory distress syndrome, which has led to one-third of patients hospitalized with COVID-19 presenting with respiratory failure [4]. The mechanisms behind COVID-associated damage to alveoli have been extensively studied in recent years [5,6,7,8,9,10]. The recruitment and activation of immune cells are considered the driving forces behind inflammation in the lung [11]. Cytokines, and especially chemokines, are deeply involved in the chemoattraction, proliferation, and activation of immune effector cells within the inflammatory focus in the alveolar tissue [12]. Current chemokine classification is based on amino acid arrangement—specifically, on the placement of cysteine—which allows us to divide the existing chemokines into four subfamilies: CC, CXC, CX3C, and XC. Chemokines and their receptors connect into a network and are mainly known for their role in controlling cell migration. However, chemokines are involved in numerous processes, including the activation and co-stimulation of cells, angio- and embryogenesis, phagocytosis, apoptosis, and many more [13].

Chemokines control all the processes behind cell trafficking; therefore, a study of chemokines in individual patients with different COVID-19 presentations may be an interesting contribution to current investigations of the mechanisms that lead to lung tissue damage. It is, however, crucial to take into account the important factor of mutations within the virion, which can potentially affect the clinical course of SARS-CoV-2 infection. As of June 2022, the World Health Organization (WHO) has been tracking two so-called ‘variants of concern’, Delta and Omicron, but since the beginning of the pandemic there have been over 13 variants detected in different parts of the world [14]. Notably, disease severity and manifestation vary depending on the genetic variant, especially for the Omicron variant [15,16,17], which may be due to the mutations causing changes in the viral proteins. By changing its antigenic structure, the SARS-CoV-2 virion manages to avoid immune control in the lung, thus lowering the inflammatory reactivity in tissues [18]. Therefore, the force of the activation and recruitment of immune cells may also vary drastically in patients infected with different SARS-CoV-2 variants.

By uniting two of the above-mentioned factors (the chemokine profile and the SARS-CoV-2 variants), we established as our objective the measurement and assessment of the different concentrations of chemokines involved in the infectious process of COVID-19 caused by different variants of the virus.

## 2. Results

### 2.1. The Chemokine Profile in Patients Infected with the Ancestral Wuhan Variant

**Figure 1:**
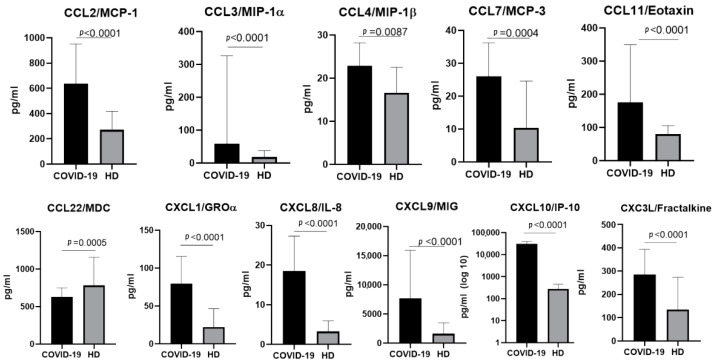
A comparison of the concentrations of the chemokines in the plasma of individuals infected with the original strain of SARS-CoV-2 in the acute stage (COVID-19), *n* = 56, and in healthy donors (HD), *n* = 51. The concentrations are presented in pg/mL plots representing medians with interquartile ranges. For CXCL10/IP-10, the *Y*-axis is presented as a logarithmic scale. Only chemokines with a statistically significant difference are presented.

In plasma samples taken from patients infected with the original Wuhan strain, we noted a statistically significant elevation in the concentrations of CCL2/MCP-1, CCL3/MIP-1α, CCL4/MIP-1β, CCL7/MCP-3, CCL11/Eotaxin, CXCL1/GROα, CXCL8/IL-8, CXCL9/MIG, CXCL10/IP-10, and CX3CL1/Fractalkine, as well as an enormous increase in the concentration of CXCL10/IP10. Notably, the concentration of one of the chemokines (CCL22/MDC) was lower in patients with acute COVID-19 than in healthy donors (Figure 1).

### 2.2. The Chemokine Profile in Patients Infected with the Alpha Variant

**Figure 2:**
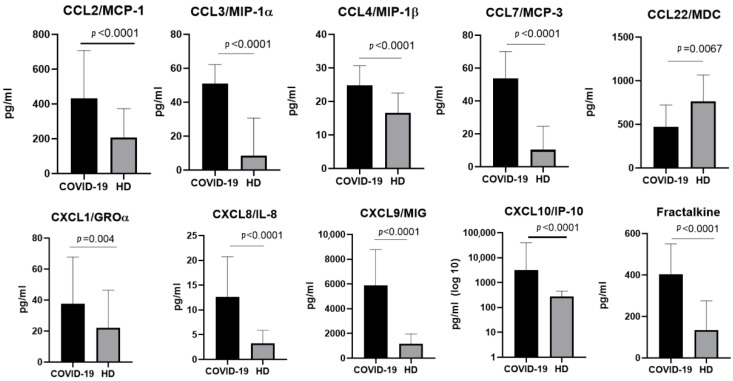
A comparison of the concentrations of the chemokines in the plasma of individuals infected with the Alpha variant of SARS-CoV-2 in the acute stage (COVID-19), *n* = 95, and in healthy donors (HD), *n* = 51. The concentrations are presented in pg/mL plots representing medians with interquartile ranges. Only chemokines with a statistically significant difference are presented.

In samples taken from patients infected with the Alpha SARS-CoV-2 variant, we observed statistically significant increases in the concentrations of CCL2/MCP-1, CCL3/MIP-1α, CCL4/MIP-1β, CCL7/MCP-3, CXCL1/GROα, CXCL8/IL-8, CXCL9/MIG, CXCL10/IP-10, and CX3CL1/Fractalkine. For CCL22/MDC, the concentrations in the healthy donors were higher than in patients infected with SARS-CoV-2 (Figure 2).

### 2.3. The Chemokine Profile in Patients Infected with the Delta Variant

**Figure 3:**
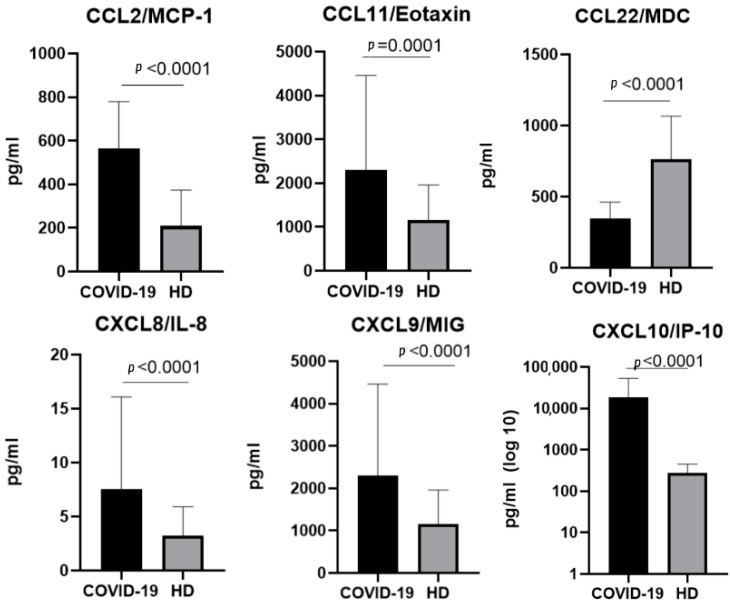
A comparison of the concentrations of the chemokines in the plasma of individuals infected with the Delta variant of SARS-CoV-2 in the acute stage (COVID-19), *n* = 78 and in healthy donors (HD), *n* = 51. The concentrations are presented in pg/mL plots representing medians with interquartile ranges. Only chemokines with a statistically significant difference are presented.

For the Delta variant, 6 of the 11 chemokines showed statistically significant changes in comparison with samples taken from healthy donors (CCL2/MCP-1, CCCL11/Eotaxin, CCL22/MDC, CXCL8/IL-8, CXCL9/MIG, and CXCL10/IP-10). For CCL22/MDC, the concentrations in the plasma samples were higher in patients with acute COVID-19 infection (Figure 3).

### 2.4. The Chemokine Profile in Patients Infected with the Omicron Variant

**Figure 4:**
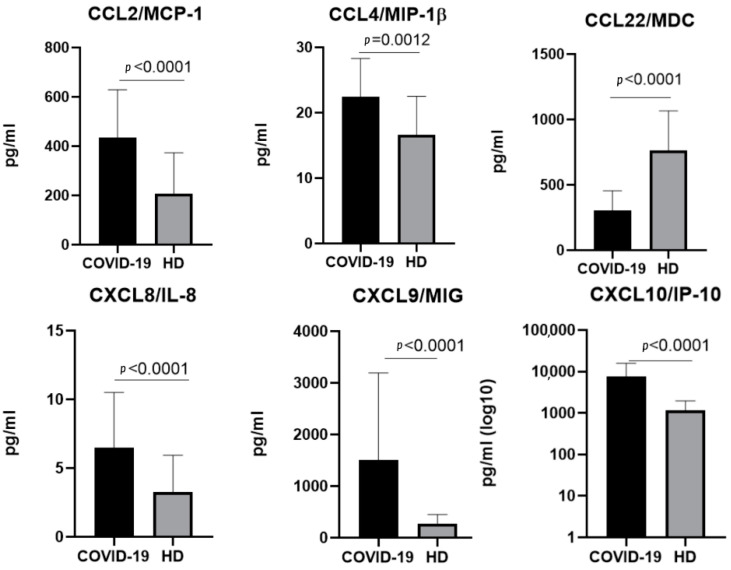
A comparison of the concentrations of the chemokines in the plasma of individuals infected with the Omicron variant of SARS-CoV-2 in the acute stage (COVID-19), *n* = 57, and in the healthy donors (HD), *n* = 51. The concentrations are presented in pg/mL plots representing medians with interquartile ranges. Only chemokines with a statistically significant difference are presented. Additional data on the differences in the chemokine concentrations for each variant can be found in Supplemental Tables S1–S4.

In samples taken from patients infected with the Omicron variant, statistically significant differences between groups were noticed for the concentrations of CCL2/MCP1, CCL4/MIP-1β, CCL22/MDC, CXCL8/IL-8, CXCL9/MIG, and CXCL/IP-10 (Figure 4).

### 2.5. A Comparison of the Chemokines between the Variants

When comparing the chemokine profiles of patients with different genetic variants of COVID-19, we divided the cohorts in groups according to the severity of the disease (moderate and severe) due to the heterogeneity in the clinical course of infection caused by different variants.

**Figure 5:**
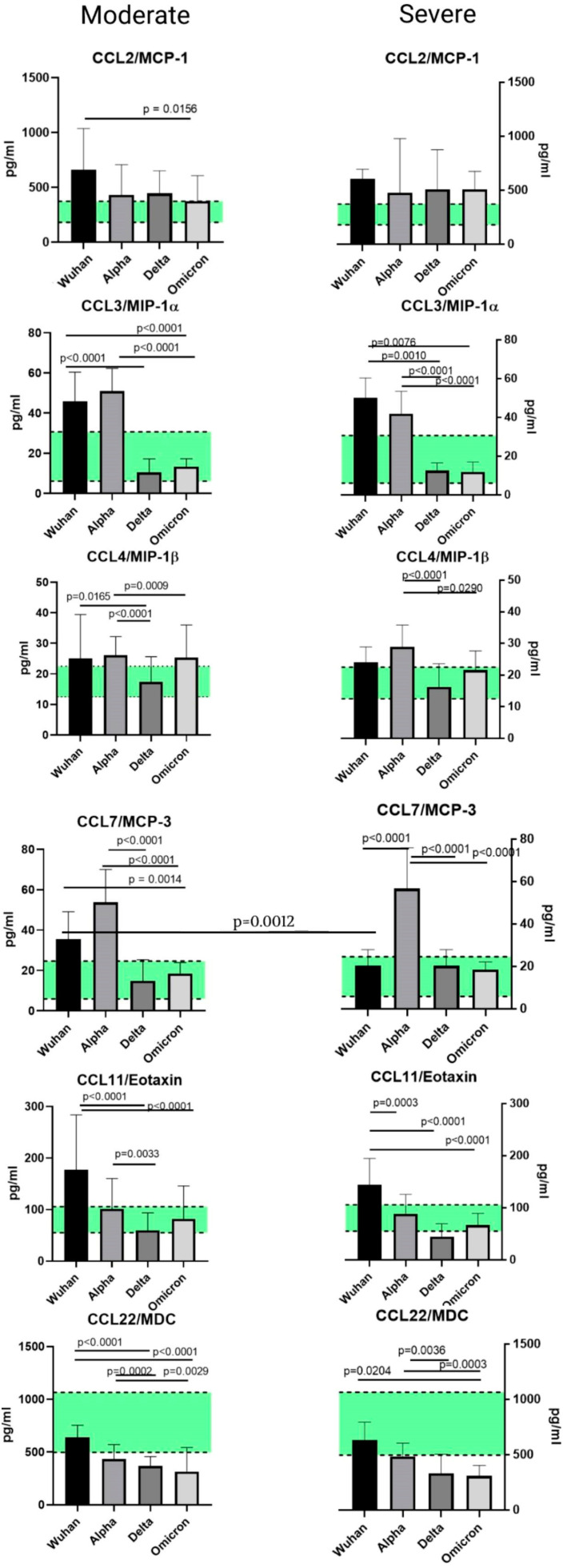
A comparison of the concentrations of C-C chemokines in patients with Wuhan, Alpha, Delta, and Omicron strains, depending on severity. The concentrations are presented in pg/mL (median and interquartile range). The green stripe represents Q25–Q75 of the chemokine concentrations in the samples from healthy donors.

**Figure 6:**
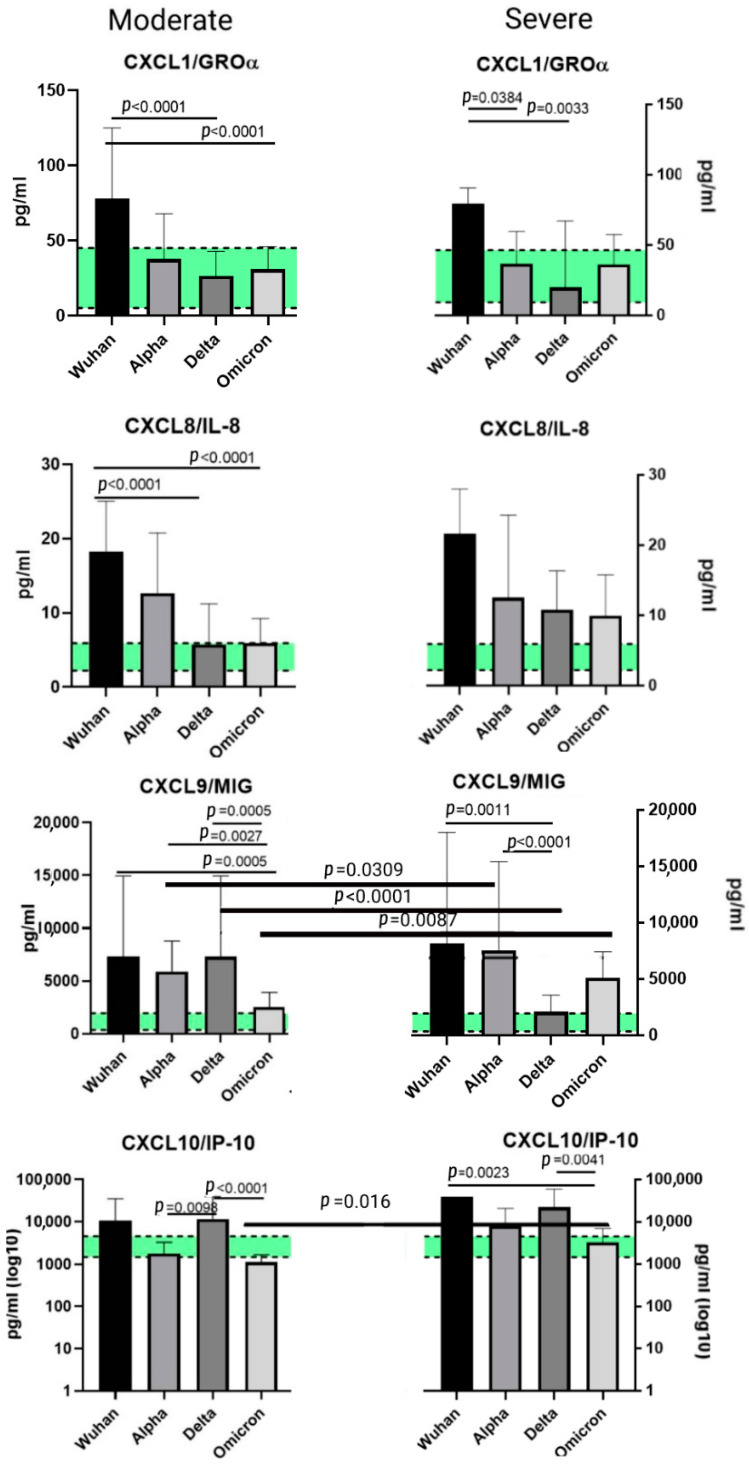
A comparison of the concentrations of C-X-C chemokines in patients with Wuhan, Alpha, Delta, and Omicron strains, depending on severity. The concentrations are presented in pg/mL (median and interquartile range). For CXCL10/IP-10, the concentration axis (Y) is presented as a logarithmic (log10). The green stripe represents Q25–Q75 of the chemokine concentrations in the samples from healthy donors.

**Figure 7:**
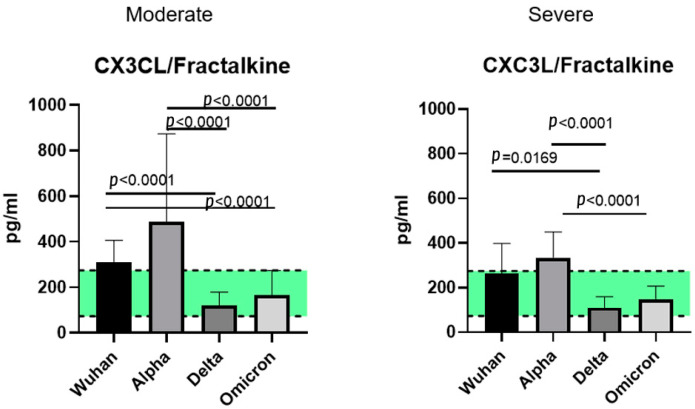
A comparison of the concentrations of C-X-3 chemokine Fractalkine in patients with Wuhan, Alpha, Delta, and Omicron strains, depending on severity. The concentrations are presented in pg/mL (median and interquartile range). The green stripe represents Q25–Q75 of the chemokine concentrations in the samples from healthy donors.

We excluded patients with mild COVID-19 for the Wuhan variant for this analysis due to the fact that cohorts infected with other variants did not include patients with this level of infection severity. When comparing the samples from the patients with different infection severities of the same genetic variant, we noted differences in the CCL7/MCP-3, CXCL9/MIG, and CXCL10/IP-10 concentrations. Between the variants, differences in concentrations were noted for MCP-1 (Wuhan and Omicron strains), CXCL1/GROα, CXCL8/IL-8 (Wuhan–Delta, Wuhan–Omicron comparisons), CCL3/MIP-1α, CCL4/MIP-1β, CCL7/MCP-3, CCL11/Eotaxin, CCL22/MDC, CXCL9/MIG, and CXCL10/IP-10 (for all the abovementioned variants). The comparisons between the variants are presented in Figure 5, Figure 6 and Figure 7.

## 3. Discussion

The question of the differences in clinical courses—specifically, in disease severity and outcome—between the different SARS-CoV-2 variants has been studied extensively [18,19,20,21,22]. By undergoing mutations in genes responsible for the expression of viral proteins (specifically, spike and nucleocapsid proteins), the SARS-CoV-2 virion changes its properties, e.g., its ability to infect new cells, to further reproduce, and to evade host immune responses [23,24]. Therefore, studying the clinical manifestations of each variant separately and comparing them is a priority for understanding and overcoming COVID-19 as a threat. When comparing chemokine concentrations between variants within cohorts of the same severity, we noted many differences between the variants.

### 3.1. Chemokines of the C-C Subfamily

#### 3.1.1. CCL2/MCP-1

Monocyte chemoattractant protein-1 (MCP-1) is a chemokine of the C-C subfamily that originates from fibroblasts and endothelial, epithelial, smooth muscle, mesangial, astrocytic, monocytic, and microglial cells. It attracts the immune cells (monocytes and basophils, memory T-cells, and NK cells) to the damaged tissues, ensuring antiviral protection. It is known to clear the SARS-CoV-2 viral load in the absence of other immune factors (CD4+, CD8+ cells) after 12 days of infection [25]. One of the roles of CCL2/MCP-1 seems to be the trafficking of monocytes to the stressed and damaged vascular endothelium. When comparing the different variants of SARS-CoV-2, we noticed that the only statistically significant difference in concentration belonged to the patients infected with the Wuhan or Omicron variants, and specifically those who were moderately ill at the time of admission. Interestingly, the concentrations of CCL2/MCP-1 were higher than those in the healthy donors for all variants, but less so for the Omicron variant. This finding may be due to the fact that the Omicron variant tends to present itself in milder forms compared to other variants [26]. However, a severe course of the Omicron variant is usually characterized by the same symptoms as the other variants of concern. CCL2/MCP-1 showed elevated concentrations compared to the healthy donors, independently of the variant of the virus. 

#### 3.1.2. CCL3/MIP-1α and CCL4/MIP-1β

Macrophage inflammatory proteins (MIP-1α and MIP-1β) belong to the C-C subfamily. These chemokines can be synthesized by most mature hematopoietic cells, i.e., monocytes, T lymphocytes, B lymphocytes, neutrophils, dendritic cells, and natural killer cells. However, under normal circumstances, the secretion of these chemokines is low until a triggering factor (usually an infectious agent and/or co-stimulation by other cells) is introduced [27]. The role of these MIP chemokines is to attract macrophages to the inflammatory foci. They also participate in wound healing [28].

In our cohorts, the levels of MIP chemokines CCL3/MIP-1α and CCL4/MIP-1β, although exceeding the numbers found in the healthy donors’ cohort, varied depending on the COVID-19 severity and virus genetic variant. The highest concentration of MIP-1α was noted in patients with the severe Wuhan strain or the moderate Alpha strain. The lowest concentration of this chemokine was detected in patients with the Delta strain, and a similar observation was made for CCL4/MIP-1β. The Delta strain is seemingly characterized by a less active production of the MIP family in comparison with other variants. When we compared the concentrations of this chemokine family in separate variants, independently of severity, we noted a statistically significant elevation in MIP-1β in the patients infected with the Alpha strain.

#### 3.1.3. CCL7/MCP-3

MCP-3 (monocyte chemoattractant protein 3), or chemokine CCL7, is part of the C-C subfamily. Under physiological conditions, it is widely expressed in body cells, e.g., in stromal cells, myocytes of the respiratory tract, and keratinocytes. Its secretion is also present in tumor cells. CCL7/MCP-3 is a potent chemoattractant for leukocytes, including monocytes, eosinophils, basophils, dendritic cells (DCs), NK cells, and activated T-lymphocytes. Similarly to CCL2/MCP-1, it attracts leukocytes to the focus of infection [29].

When comparing CCL7/MCP-3 levels in plasma, we noted a significant elevation in this chemokine concentration in the Alpha variant in contrast to other variants. When comparing this chemokine in separate cohorts with healthy donors, we observed higher concentrations of CCL7/MCP-3 in the patients with the original SARS-CoV-2 Wuhan strain and the Alpha variant.

#### 3.1.4. CCL11/Eotaxin

CCL11/Eotaxin also belongs to the subfamily of C-C chemokines; it plays a major role in eosinophilic inflammation, which is common in asthma. It is constitutively expressed only in adult intestine, thymus, and skin. Eosinophils are a source of cytotoxic granular proteins responsible for the tissue damaging and remodeling implicated in the physiopathology of several diseases such as asthma [30]. Of the four variants, CCL11/Eotaxin levels were the highest in patients with the original Wuhan strain, with both moderate and severe infection. When we investigated the CCL11/Eotaxin concentrations for each variant and compared them with healthy donors, we did not find any statistically significant differences.

#### 3.1.5. CCL22/MDC

Macrophage-derived chemokine (MDC) is responsible for the attraction of multiple cell types, including DC, NK, T-helper cells, and cytotoxic cells. Interestingly, it was believed to be a chemokine of constitutional secretion—a theory now disproven [31]. In our study, we noticed an interesting trend concerning CCL22/MDC in COVID-19 pathogenesis: namely, a decrease in the concentrations of this chemokine in comparison with healthy donors. This trend was present for all four SARS-CoV-2 variants. When we analyzed other studies on this topic, we noted that, in lung pathologies of other origins (i.e., eosinophilic pneumonia and pulmonary hemorrhage), CCL22/MDC concentrations were significantly higher than in healthy donors [32,33]. In COVID-19 infection, however, the CCL22/MDC secretion seems to be suppressed. These data are preliminarily supported by the findings of other researchers [34]; however, for the study in question, the genetic testing of the SARS-CoV-2 genome was not performed. The lower levels of CCL22/MDC in comparison with healthy donors raises the question of the potential depletion of this chemokine mid-infection, or the involvement of CCL22/MDC in the viral cycle of SARS-CoV2.

### 3.2. Chemokines of the C-X-C Subfamily

#### 3.2.1. CXCL1/GROα

CXCl1/GROα is produced by a range of immune cells such as macrophages, neutrophil and epithelial cells, and T-helper cells—specifically, Th17 cells [35,36]. It specializes in neutrophil recruitment to the infection and/or damaged tissue site, and is deeply involved in cancer development. When comparing variants in the samples from patients with severe infection, we observed an increase in concentration typical for the Wuhan strain. Higher levels of plasma CXCL1/GROα have been previously reported in patients with severe original strains of SARS-CoV-2 [37,38]. When comparing each variant cohort with the healthy donors, we noted an increase in concentrations in the plasma samples from patients infected with the original Wuhan strain and those infected with the Alpha variant.

#### 3.2.2. CXCL8/IL-8

The elevation in CXCL8/IL-8 in COVID-19 patients is a common finding supported by multiple studies. This chemokine is often recognized as one of the potential biomarkers of infection severity, although its role in the pathogenesis of SARS-CoV-2-induced infection is yet to be explored, since CXCL8/IL-8 is mostly associated with neutrophil trafficking [30,39,40]. In our study, we noted a statistically significant elevation in CXCL8/IL-8 for all variants. When comparing the levels of CXCL8/IL-8 in patients with a moderate clinical presentation of the infection, we noted a statistically significant elevation in concentrations in the plasma samples of patients infected with the Wuhan strain. In comparison to healthy donors, CXCL8/IL-8 elevation was observed for patients independent of the variant.

#### 3.2.3. CXCL9/MIG

Monokine induced by gamma interferon (MIG) is the chemokine that belongs to the so-called group of ‘pro-inflammatory’ chemokines [41]. It is secreted upon immune stimuli and is not constantly present in plasma. Lower levels of CXCL9/MIG are associated with a negative outcome of COVID-19 infection due to the fact that CXCL9/MIG is involved in Th1 immune responses. When the latter is insufficient, the secretion of CXCL9/MIG is also reduced [42]. In our study, we observed lower concentrations of CXCL9/MIG in patients with severe Delta infection. When compared to the cohort of healthy donors, CXCL9/MIG concentrations were higher in the samples taken from patients infected with the Wuhan, Alpha and Omicron variants.

#### 3.2.4. CXCL10/IP-10

Interferon gamma inducible protein-10 (IP-10) is secreted in response to IFN-γ by monocytes, endothelial cells, and fibroblasts. This chemokine has been identified as one of the four primary cytokines that play a part in the so-called ‘cytokine storm’, hyperinflammation in response to present SARS-CoV-2 infection, and thrombosis [43]. In our study, we noted a significant elevation in CXCL10/IP-10 in COVID-infected patients compared to healthy donors. Interestingly, elevation varied depending on the genetic variant of the virus—for instance, in severely ill patients with the Wuhan strain, the concentrations of CXCL10/IP-10 were nearly 40 times higher than those in patients infected with the Omicron variant. In general, CXCL10/IP-10 plasma concentration exhibited an upsurge compared to healthy donors—a tendency noted in all variants. This increase in CXCL/IP-10 is supported by previous studies, and it is currently being investigated not only as a biomarker of disease severity but also as a potential target for therapy. Intercepting the IFN-γ-associated activation of JAK-STAT and the following cytokine release (including the release of CXCL10/IP-10) may prove to be a promising strategy to inhibit the cytokine storm [44].

### 3.3. CX3C Family Chemokines: CX3CL1/Fractalkine

CXC3L1 chemokine, or Fractalkine, is a large chemokine that can bind to the cellular surfaces of the endothelium due to its unique structure. However, CXC3L1/Fractalkine also exists in a soluble form, attracting various T-lymphocytes and monocytes [43]. Some studies have proposed a theory of CXC3L1/Fractalkine involvement in COVID-associated thrombosis [45]. Although the concentrations of CXC3L1/Fractalkine varied depending on the variant, we did not observe any statistically significant elevations in cohorts compared to healthy donors. Therefore, the role of CXC3L1/Fractalkine in COVID-induced immune responses is yet to be investigated.

## 4. Materials and Methods

### 4.1. Samples

Our longitudinal study was performed in the timespan between May 2020 and April 2022. As a substrate for detection of chemokine concentrations, we used the biological samples (blood plasma) of patients infected with different variants of SARS-CoV-2, i.e., the ancestral Wuhan strain (56 samples collected from patients hospitalized in May 2020), the Alpha variant (95 samples collected from patients hospitalized in October 2020), the Delta variant (78 samples collected from patients hospitalized between August and November 2021), and the Omicron variant (57 samples collected from patients hospitalized between February and April 2022). 

All the samples (*n* = 317) were taken from patients with PCR-verified COVID-19. All the patients were hospitalized with an official diagnosis of COVID-19 at two hospitals in Saint Petersburg, Russia: a COVID-19-specialized hospital, Pavlov First Saint Petersburg State Medical University, and the North-Western Scientific and Clinical Center named after L.G. Sokolov.

We also included samples from healthy donors (*n* = 51) in our study.

Whole blood samples were collected in vacuum tubes with EDTA anticoagulant, followed by centrifugation (350 g for 10 min) and plasma extraction. Plasma samples were transferred to cryotubes and frozen at −80 °C prior to analysis. 

### 4.2. Patients

The patients were recruited to the study at the time of admission, and their blood was taken within the first 7–10 days from the onset of infection. The selection criteria were: age above 18 years old, a positive COVID-19 test, and a clinically confirmed diagnosis without known previous SARS-CoV-2 infection and/or vaccination. Patients were excluded from the study if they had any diagnosed immunodeficiencies, co-infections, or comorbidities in an active stage, including pregnancy. We also excluded patients who, prior to their hospital admission, were treated with something other than occasional non-steroid anti-inflammatory drugs to lower their fever. Therefore, patients who received antiviral and steroid drugs were not included in the study.

The demographics slightly varied for cohorts infected with different variants; elaborated data on this topic can be found in Table 1.

All the infected patients were diagnosed with COVID-19 (U07.1) at the hospitals, with the virus identified by qualitative PCR (via the detection of SARS-CoV-2 RNA). All individuals in this cohort presented with typical COVID-19-associated symptoms, i.e., fever, a sense of fatigue, muscle and joint pains, a cough, and pneumonia confirmed by CT-scans. 

Disease severity was assessed by the medical staff of the hospitals based on the guidelines on COVID-19 infection provided by the Russian Ministry of Healthcare, which followed WHO guidelines [46]. Among the criteria for determining disease severity were age, oxygen saturation, chest imaging (e.g., CT), and other clinical symptoms for determining the severity of pneumonia.

The severity of COVID-19 varied within groups depending on the virus variant (Table 2).

The study’s protocol was approved by the ethics committee of the Saint Petersburg Pasteur Institute in accordance with the Declaration of Helsinki. All participants were informed of our study and willingly signed consent forms.

### 4.3. Genotyping of the SARS-CoV-2 Genetic Variants

The genotyping of SARS-CoV-2 isolates collected from patients was performed based on near-complete genome sequences using the Illumina MiSeq automatic platform (Illumina Inc., San Diego, CA, USA). Nasopharyngeal swabs from COVID-19 patients were studied previously using the COVID-19 Amp RT-qPCR Kit (Saint Petersburg Pasteur Institute, Saint Petersburg, Russian Federation) according to the manufacturer’s recommendations for SARS-CoV-2 detection and concentration assessment. Swabs were collected in 500 µL of a special transport medium (AmpliSens^®^, Moscow, Russian Federation) or phosphate-buffered saline (pH 7.0) and stored at −20 °C until analysis. Total nucleic acid samples were obtained by extraction and purification using the RIBO-prep DNA/RNA Extraction Kit (AmpliSens^®^, Moscow, Russian Federation) according to the manufacturer’s recommendations. DNA/RNA was eluted with 50 µL of the elution buffer (AmpliSens^®^, Moscow, Russian Federation) and stored at −70 °C until molecular analysis. Reverse transcription was performed using random hexanucleotide primers and the Reverta-L Kit (AmpliSens^®^, Moscow, Russia) according to the manufacturer instructions; cDNA samples were stored at −70 °C and subsequently used as amplification templates. Libraries were prepared using the TruSeq Nano DNA Kit (Illumina Inc., San Diego, CA, USA) and the TruSeq DNA CD Indexes Kit (Illumina Inc., San Diego, CA, USA). Quality assessment of the final libraries was carried out on the QIAxcel Advanced capillary system (Qiagen, Germany). Sequencing was performed using the Illumina MiSeq System (Illumina Inc., San Diego, CA, USA) with the MiSeq Reagent Kit v3 (600-cycle) (Illumina Inc., San Diego, CA, USA).

### 4.4. Chemokine Detection

To measure the concentrations of chemokines, we used xMAP multiplexing technology for Luminex MagPix (Austin, TX, USA). It uses labeled beads for the simultaneous capture of multiple analytes. For our study, we used the Millipore^®^ kit (Burlington, MA, USA) for the detection of 47 cytokines/chemokines. Among analytes detectable with the kit, there are 11 chemokines (CCL2/MCP-1, CCL3/MIP-1α, CCL4/MIP-1β, CCL7/MCP-3, CCL11/Eotaxin, CCL22/MDC, CXCL1/GROα, CXCL8/IL-8, CXCL9/MIG, CXCL10/IP-10, CX3CL1/Fractalkine).

### 4.5. Statistical Analysis

For the comparison of multiple data groups, the Kruskal–Wallis test was used with an additional Dunn’s test for multiple comparisons. For two-group analysis, we used the Mann–Whitney U-test for continuous variables. When interpreting statistical analysis results, we designated *p* < 0.01 to be statistically significant

Data analysis was performed in GraphPad Prism 8.0 and the SPSS Statistics software. Data visualization was conducted via GraphPad Prism 8.0 and Microsoft Excel 2013 graphing tools. 

## 5. Conclusions

Chemokine assessments for each variant performed separately from each other revealed several patterns in the COVID-19-induced chemokine profile in comparison with healthy donors:

1. A statistically significant elevation in CCL2/MCP-1 concentrations in patients with COVID-19, independently from the variant. This tendency can be explained by the role MCP-1 plays in the attraction of monocytes to the sites of damaged vascular endothelium.
2. A prominent increase in CXCL1/IP-10 levels in all the COVID-infected cohorts in comparison with healthy donors. IP-10 is often recognized as one of the markers of the cytokine storm; it is deeply involved in thrombosis and ARDS in patients with COVID-19.
3. A statistically significant rise in CXCL8/IL-8 concentrations in patients with COVID-19, independently from the detected variant. This is a common finding in COVID-19 infection, and IL-8 is often recognized as a severity marker.
4. A drop in the concentrations of CCL22/MDC in infected patients compared to healthy donors. In lung damage associated with other etiological factors (for instance, eosinophilic pneumonia or pulmonary hemorrhage), macrophage-derived chemokine was present in higher concentrations in plasma than it was in healthy donors. A decrease in the concentrations of this chemokine may be related to the life cycle of SARS-CoV-2. The severity of the disease is negatively correlated with the concentrations of MDC.

When comparing the different variants of SARS-CoV-2, we found it noteworthy that out of all the investigated chemokines, only three showed stability in terms of a lack of differences between the variants: CCL2/MCP-1, CXCL1/IP-10, CXCL8/IL-8, and CCL22/MDC. Our study is the first to highlight the differences in the chemokine profile among genetic variants of SARS-CoV-2. Overall, the concentrations of the chemokines vary significantly depending on the variant of the virus, leading us to infer that mutations in viral proteins play a role not only in terms of viral properties but also in the pathogenesis of the infection and the cellular and molecular mechanisms of immune responses. While some chemokines can potentially be used as biomarkers of disease severity, or even as targets for anti-inflammatory blockage, it is also important to study their role in antiviral clearance.

## Figures and Tables

**Table 1:** Characterization of patients and healthy donors’ cohort.

Variant	Wuhan (*n* = 56)	Alpha (*n* = 95)	Delta (*n* = 78)	Omicron (*n* = 57)	Healthy Donors (*n* = 51)
Age, years (Me; Q25, Q75)	66 (32; 75)	72 (28; 84)	71 (45; 93)	69 (32; 91)	49 (24; 69)
Gender distribution	50% female (*n* = 28); 50% male (*n* = 28)	52.7% female (*n* = 50); 47.3% male (*n* = 45)	64.1% female (*n* = 50); 35.9% male (*n* = 28)	61.4% female (*n* = 35); 38.6% male (*n* = 22)	58.8% female (*n* = 30), 41.2% male (*n* = 21)

**Table 2:** Disease severity within cohorts.

Variant	% of Patients, Diagnosed with ‘Mild’ Infection	% of Patients, Diagnosed with ‘Moderate’ Infection	% of Patients, Diagnosed with ‘Severe’ Infection
Ancestral Wuhan	43.5% (*n* = 30)	46.4% (*n* = 32)	10.1% (*n* = 7)
Alpha	0	26.6% (*n* = 25)	73.6% (*n* = 70)
Delta	0	34.6% (*n* = 27)	65.4% (*n* = 51)
Omicron	0	75.4% (*n* = 43)	24.6% (*n* = 14)

## Data Availability

Data sharing not applicable.

## Supplementary Materials

The supporting information can be downloaded at: https://www.mdpi.com/article/10.3390/ijms23169058/s1.

## References

[1] Wang X.-M., Mannan R., Xiao L., Abdulfatah E., Qiao Y., Farver C., Myers J.L., Zelenka-Wang S., McMurry L., Su F. (2021). Characterization of SARS-CoV-2 and host entry factors distribution in a COVID-19 autopsy series. Commun. Med..

[2] Ozono S., Zhang Y., Ode H., Sano K., Tan T.S., Imai K., Miyoshi K., Kishigami S., Ueno T., Iwatani Y. (2021). SARS-CoV-2 D614G spike mutation increases entry efficiency with enhanced ACE2-binding affinity. Nat. Commun..

[3] Samavati L., Uhal B.D. (2020). ACE2, Much More Than Just a Receptor for SARS-COV-2. Front. Cell. Infect. Microbiol..

[4] Attaway A.H., Scheraga R.G., Bhimraj A., Biehl M., Hatipoğlu U. (2021). Severe COVID-19 pneumonia: Pathogenesis and clinical management. BMJ.

[5] D’Agnillo F., Walters K.-A., Xiao Y., Sheng Z.-M., Scherler K., Park J., Gygli S., Rosas L.A., Sadtler K., Kalish H. (2021). Lung epithelial and endothelial damage, loss of tissue repair, inhibition of fibrinolysis, and cellular senescence in fatal COVID-19. Sci. Transl. Med..

[6] Rendeiro A.F., Ravichandran H., Bram Y., Chandar V., Kim J., Meydan C., Park J., Foox J., Hether T., Warren S. (2021). The spatial landscape of lung pathology during COVID-19 progression. Nature.

[7] Szabo P.A., Dogra P., Gray J.I., Wells S.B., Connors T.J., Weisberg S.P., Krupska I., Matsumoto R., Poon M.M., Idzikowski E. (2021). Longitudinal profiling of respiratory and systemic immune responses reveals myeloid cell-driven lung inflammation in severe COVID-19. Immunity.

[8] Suran M. (2021). Autopsies Reveal Lung Damage Patterns From COVID-19. JAMA.

[9] Upadhya S., Rehman J., Malik A.B., Chen S. (2022). Mechanisms of lung injury induced by SARS-CoV-2 infection. Physiology.

[10] Kommoss F.K., Schwab C., Tavernar L., Schreck J., Wagner W.L., Merle U., Jonigk D., Schirmacher P., Longerich T. (2020). The Pathology of Severe COVID-19-Related Lung Damage. Dtsch. Arztebl. Int..

[11] Stenmark K.R., Frid M.G., Gerasimovskaya E., Zhang H., McCarthy M.K., Thurman J.M., Morrison T.E. (2021). Mechanisms of SARS-CoV-2-induced lung vascular disease: Potential role of complement. Pulm. Circ..

[12] Matthay M.A., Leligdowicz A., Liu K.D. (2020). Biological Mechanisms of COVID-19 Acute Respiratory Distress Syndrome. Am. J. Respir. Crit. Care Med..

[13] Hughes C.E., Nibbs R.J.B. (2018). A guide to chemokines and their receptors. FEBS J..

[14] World Health Organization (WHO) Tracking SARS-CoV-2 Variants. https://www.who.int/en/activities/tracking-SARS-CoV-2-variants/.

[15] Danchin A., Timmis K. (2020). SARS-CoV-2 variants: Relevance for symptom granularity, epidemiology, immunity (herd, vaccines), virus origin and containment?. Environ. Microbiol..

[16] Menni C., Valdes A.M., Polidori L., Antonelli M., Penamakuri S., Nogal A., Louca P., May A., Figueiredo J.C., Hu C. (2022). Symptom prevalence, duration, and risk of hospital admission in individuals infected with SARS-CoV-2 during periods of omicron and delta variant dominance: A prospective observational study from the ZOE COVID Study. Lancet.

[17] Abdullah F., Myers J., Basu D., Tintinger G., Ueckermann V., Mathebula M., Ramlall R., Spoor S., de Villiers T., Van der Walt Z. (2021). Decreased severity of disease during the first global omicron variant COVID-19 outbreak in a large hospital in Tshwane, South Africa. Int. J. Infect. Dis..

[18] Elizondo V., Harkins G.W., Mabvakure B., Smidt S., Zappile P., Marier C., Maurano M.T., Perez V., Mazza N., Beloso C. (2021). SARS-CoV-2 genomic characterization and clinical manifestation of the COVID-19 outbreak in Uruguay. Emerg. Microbes Infect..

[19] Nagy Á., Pongor S., Győrffy B. (2020). Different mutations in SARS-CoV-2 associate with severe and mild outcome. Int. J. Antimicrob. Agents.

[20] Al Khatib H.A., Benslimane F.M., Elbashir I.E., Coyle P.V., Al Maslamani M.A., Al-Khal A., Al Thani A.A., Yassine H.M. (2020). Within-Host Diversity of SARS-CoV-2 in COVID-19 Patients With Variable Disease Severities. Front. Cell. Infect. Microbiol..

[21] Hoang V.-T., Colson P., Levasseur A., Delerce J., Lagier J.-C., Parola P., Million M., Fournier P.-E., Raoult D., Gautret P. (2021). Clinical outcomes in patients infected with different SARS-CoV-2 variants at one hospital during three phases of the COVID-19 epidemic in Marseille, France. Infect. Genet. Evol..

[22] Cao C., He L., Tian Y., Qin Y., Sun H., Ding W., Gui L., Wu P. (2021). Molecular epidemiology analysis of early variants of SARS-CoV-2 reveals the potential impact of mutations P504L and Y541C (NSP13) in the clinical COVID-19 outcomes. Infect. Genet. Evol..

[23] Harvey W.T., Carabelli A.M., Jackson B., Gupta R.K., Thomson E.C., Harrison E.M., Ludden C., Reeve R., Rambaut A., COVID-19 Genomics UK (COG-UK) Consortium (2021). SARS-CoV-2 variants, spike mutations and immune escape. Nat. Rev. Microbiol..

[24] Eaaswarkhanth M., Al Madhoun A., Al-Mulla F. (2020). Could the D614G substitution in the SARS-CoV-2 spike (S) protein be associated with higher COVID-19 mortality?. Int. J. Infect. Dis..

[25] Khalil B.A., Elemam N.M., Maghazachi A.A. (2021). Chemokines and chemokine receptors during COVID-19 infection. Comput. Struct. Biotechnol. J..

[26] Jansen L., Tegomoh B., Lange K., Showalter K., Figliomeni J., Abdalhamid B., Iwen P.C., Fauver J., Buss B., Donahue M. (2021). Investigation of a SARS-CoV-2 B.1.1.529 (Omicron) Variant Cluster—Nebraska, November–December 2021. MMWR. Morb. Mortal. Wkly. Rep..

[27] Bhavsar I., Miller C.S., Al-Sabbagh M. (2015). Macrophage Inflammatory Protein-1 Alpha (MIP-1 alpha)/CCL3: As a Biomarker. Gen. Methods Biomark. Res. Appl..

[28] Menten P., Wuyts A., Van Damme J. (2002). Macrophage inflammatory protein-1. Cytokine Growth Factor Rev..

[29] Chen L., Wang G., Tan J., Cao Y., Long X., Luo H., Tang Q., Jiang T., Wang W., Zhou J. (2020). Scoring cytokine storm by the levels of MCP-3 and IL-8 accurately distinguished COVID-19 patients with high mortality. Signal Transduct. Target. Ther..

[30] Chi Y., Ge Y., Wu B., Zhang W., Wu T., Wen T., Liu J., Guo X., Huang C., Jiao Y. (2020). Serum Cytokine and Chemokine Profile in Relation to the Severity of Coronavirus Disease 2019 in China. J. Infect. Dis..

[31] Mantovani A., A Gray P., Van Damme J., Sozzani S. (2000). Macrophage-derived chemokine (MDC). J. Leukoc. Biol..

[32] Manabe K., Nishioka Y., Kishi J., Inayama M., Aono Y., Nakamura Y., Ogushi F., Bando H., Tani K., Sone S. (2005). Elevation of macrophage-derived chemokine in eosinophilic pneumonia: A role of alveolar macrophages. J. Med Investig..

[33] Richter J., Sutton J.M., Belizaire R.M., Friend L.A., Schuster R.M., Johannigman T.A., Miller S.G., Lentsch A.B., Pritts T.A. (2014). Macrophage-Derived Chemokine (CCL22) Is a Novel Mediator of Lung Inflammation Following Hemorrhage and Resuscitation. Shock.

[34] Tufa A., Gebremariam T.H., Manyazewal T., Getinet T., Webb D.-L., Hellström P.M., Genet S. (2022). Cytokine and chemokine profile in patients hospitalized with COVID-19: A comparative study. bioRxiv.

[35] Becker S., Quay J., Koren H.S., Haskill J.S. (1994). Constitutive and stimulated MCP-1, GRO alpha, beta, and gamma expression in human airway epithelium and bronchoalveolar macrophages. Am. J. Physiol. Cell. Mol. Physiol..

[36] Ma K., Yang L., Shen R., Kong B., Chen W., Liang J., Tang G., Zhang B. (2018). Th17 cells regulate the production of CXCL1 in breast cancer. Int. Immunopharmacol..

[37] Gudowska-Sawczuk M., Mroczko B. (2022). What Is Currently Known about the Role of CXCL10 in SARS-CoV-2 Infection?. Int. J. Mol. Sci..

[38] Arsentieva N.A., Liubimova N.E., Batsunov O.K., Korobova Z.R., Stanevich O.V., Lebedeva A.A., Vorobyov E.A., Vorobyova S.V., Kulikov A.N., Lioznov D.A. (2021). Plasma cytokines in patients with COVID-19 during acute phase of the disease and following complete recovery. Med. Immunol..

[39] McElvaney O.J., McEvoy N.L., McElvaney O.F., Carroll T.P., Murphy M.P., Dunlea D.M., Ni Choileain O., Clarke J., O’Connor E., Hogan G. (2020). Characterization of the Inflammatory Response to Severe COVID-19 Illness. Am. J. Respir. Crit. Care Med..

[40] Li L., Li J., Gao M., Fan H., Wang Y., Xu X., Chen C., Liu J., Kim J., Aliyari R. (2021). Interleukin-8 as a Biomarker for Disease Prognosis of Coronavirus Disease-2019 Patients. Front. Immunol..

[41] Callahan V., Hawks S., Crawford M., Lehman C., Morrison H., Ivester H., Akhrymuk I., Boghdeh N., Flor R., Finkielstein C. (2021). The Pro-Inflammatory Chemokines CXCL9, CXCL10 and CXCL11 Are Upregulated Following SARS-CoV-2 Infection in an AKT-Dependent Manner. Viruses.

[42] Tincati C., Cannizzo E.S., Giacomelli M., Badolato R., Monforte A.D., Marchetti G. (2020). Heightened Circulating Interferon-Inducible Chemokines, and Activated Pro-Cytolytic Th1-Cell Phenotype Features Covid-19 Aggravation in the Second Week of Illness. Front. Immunol..

[43] Quirch M., Lee J., Rehman S. (2020). Hazards of the Cytokine Storm and Cytokine-Targeted Therapy in Patients With COVID-19: Review. J. Med. Internet Res..

[44] Pum A., Ennemoser M., Adage T., Kungl A.J. (2021). Cytokines and Chemokines in SARS-CoV-2 Infections—Therapeutic Strategies Targeting Cytokine Storm. Biomolecules.

[45] Bazan J.F., Bacon K.B., Hardiman G., Wang W., Soo K., Rossi D., Greaves D.R., Zlotnik A., Schall T.J. (1997). A new class of membrane-bound chemokine with a CX3C motif. Nature.

[46] Rivas-Fuentes S., Valdés V.J., Espinosa B., Gorocica-Rosete P., Salgado-Aguayo A. (2021). Could SARS-CoV-2 blocking of ACE2 in endothelial cells result in upregulation of CX3CL1, promoting thrombosis in COVID-19 patients?. Med. Hypotheses.

