# Supplementary material for: A Comparative Study of the Plasma Chemokine Profile in COVID-19 Patients Infected with Different SARS-CoV-2 Variants
Source: Int J Mol Sci. 2022 Aug 13;23(16):9058. doi: 10.3390/ijms23169058 (PMC9409001; doi:10.3390/ijms23169058)
Supplement: Supplementary file 1 [file ijms-23-09058-s001.zip › ijms-1845885-supplementary.pdf]

**Table S1.** Chemokine concentrations in blood plasma samples from patients infected with ancestral Wuhan strain of SARS-CoV-2 (COVID-19), n=56; and healthy donors (HD), n=51. Concentrations presented in pg/ml.

| Chemokine | COVID-19, n = 56<br>Median (Q25-Q75) | HD, n = 51<br>Median (Q25-Q75) | COVID-19 vs. HD<br>p-value |
|---|---|---|---|
| CCL2/MCP-1 | 636.8 (458.5-951.8) | 208.5 (180.2-373.5) | <0.0001 |
| CCL3/MIP-1α | 58.8 (38.7-326.4) | 8.631 (6.1-30.7) | <0.0001 |
| CCL4/MIP-1β | 22.91 (17.2-28.2) | 16.7 (12.5-22.5) | 0.0087 |
| CCL7/MCP-3 | 26.1 (14.8-36.3) | 10.4 (6.0-24.7) | 0.0004 |
| CCL11/Eotaxin | 175.4 (121.1-355.2) | 80.61 (55.10-105.8) | <0.0001 |
| CCL22/MDC | 629.8 (513.8-749.8) | 873.5 (497.4-1265) | 0.0005 |
| CXCL1/GROα | 79.4 (55.1-115.4) | 22.2 (9.4-46.4) | <0.0001 |
| CXCL8/IL-8 | 18.5 (15.5-27.3) | 3.2 (2.2-5.9) | <0.0001 |
| CXCL9/MIG | 3985.0 (7677.1-15917.8) | 1161 (364.0-1959) | <0.0001 |
| CXCL10/IP-10 | 31238. 0 (3213.5-40000.0) | 278.1 (147.9-451.9) | <0.0001 |
| CX3CL1/Fractalkine | 286.1 (202.3-393.5) | 135.0 (73.23-274.8) | <0.0001 |

**Table S2.** Chemokine concentrations in blood plasma samples from patients infected with Alpha variant of SARS-CoV-2 (COVID-19), n=95; and healthy donors (HD), n=51. Concentrations presented in pg/ml.

| Chemokine | COVID-19, n = 95<br>Median (Q25-Q75) | HD, n = 51<br>Median (Q25-Q75) | COVID-19 vs. HD<br>p-value |
|---|---|---|---|
| CCL2/MCP-1 | 430.9 (254.3-706.8) | 208.5 (180.2-373.5) | <0.0001 |
| CCL3/MIP-1α | 51.0 (40.3-62.3) | 8.631 (6.1-30.7) | 0.0002 |
| CCL4/MIP-1β | 24.8 (21.7-30.7) | 16.7 (12.5-22.5) | <0.0001 |
| CCL7/MCP-3 | 53.8 (39.8-70.1) | 10.4 (6.0-24.7) | <0.0001 |
| CCL11/Eotaxin | 90.7 (65.8-118.6) | 80.61 (55.10-105.8) | ns |
| CCL22/MDC | 474.1 (424.2-722.2) | 873.5 (497.4-1265) | 0.0067 |
| CXCL1/GROα | 37.67 (25.7-67.8) | 22.2 (9.4-46.4) | 0.004 |
| CXCL8/IL-8 | 12.7 (7.9-20.7) | 3.2 (2.2-5.9) | <0.0001 |
| CXCL9/MIG | 5896.0 (3661.1-8790.2) | 1161 (364.0-1959) | <0.0001 |
| CXCL10/IP-10 | 3142.1 (1004.1-40000.0) | 278.1 (147.9-451.9) | <0.0001 |
| CX3CL1/Fractalkine | 404.3 (273.9-549.7) | 135.0 (73.23-274.8) | <0.0001 |

**Table S3.** Chemokine concentrations in blood plasma samples from patients infected with Delta variant of SARS-CoV-2 (COVID-19), n=78; and healthy donors (HD), n=51. Concentrations presented in pg/ml.

| Chemokine | COVID-19, n = 78 Median (Q25-Q75) | HD, n = 51 Median (Q25-Q75) | COVID-19 vs. HD p-value |
|---|---|---|---|
| CCL2/MCP-1 | 562.8 (319.0-779.3) | 208.5 (180.2-373.5) | <0.0001 |
| CCL3/MIP-1α | 11.59 (8.4-17.21) | 8.631 (6.1-30.7) | ns |
| CCL4/MIP-1β | 16.3 (12.5-20.2) | 16.7 (12.5-22.5) | ns |
| CCL7/MCP-3 | 16.8 (12.7-27.2) | 10.4 (6.0-24.7) | ns |
| CCL11/Eotaxin | 51.0 (40.0-75.3) | 80.61 (55.10-105.8) | 0.0001 |
| CCL22/MDC | 344.1 (247.2-461.9) | 873.5 (497.4-1265) | <0.0001 |
| CXCL1/GROα | 25.7 (15.5-56.9) | 22.2 (9.4-46.4) | ns |
| CXCL8/IL-8 | 7.6 (3.9-16.1) | 3.2 (2.2-5.9) | <0.0001 |
| CXCL9/MIG | 2303.0 (1232.1-4459.0) | 1161 (364.0-1959) | <0.0001 |
| CXCL10/IP-10 | 18488.0 (7084.2-53108.1) | 278.1 (147.9-451.9) | <0.0001 |
| CX3CL1/Fractalkine | 119.2 (88.2-161.8) | 135.0 (73.23-274.8) | ns |

**Table S4.** Chemokine concentrations in blood plasma samples from patients infected with Omicron variant of SARS-CoV-2 (COVID-19), n=57; and healthy donors (HD), n=51. Concentrations presented in pg/ml.

| Chemokine | COVID-19, n=57 Median (Q25-Q75) | HD, n = 51 Median (Q25-Q75) | COVID-19 ns. HD p-value |
|---|---|---|---|
| CCL2/MCP-1 | 434.6 (286.9-628.9) | 208.5 (180.2-373.5) | <0.0001 |
| CCL3/MIP-1α | 13.35 (9.5-17.3) | 8.631 (6.1-30.7) | ns |
| CCL4/MIP-1β | 22.4 (17.9-28.3) | 16.7 (12.5-22.5) | 0.0012 |
| CCL7/MCP-3 | 18.5 (13.5-23.9) | 10.4 (6.0-24.7) | ns |
| CCL11/Eotaxin | 65.2 (54.6-88.7) | 80.61 (55.10-105.8) | ns |
| CCL22/MDC | 306.1 (252.6-455.5) | 873.5 (497.4-1265) | <0.0001 |
| CXCL1/GROα | 27.33 (14.9-49.1) | 22.2 (9.4-46.4) | ns |
| CXCL8/IL-8 | 6.5 (4.3-10.5) | 3.2 (2.2-5.9) | <0.0001 |
| CXCL9/MIG | 3000.0 (1507.1-5951.2) | 1161 (364.0-1959) | <0.0001 |
| CXCL10/IP-10 | 1506.1 (592.8-3194.2) | 278.1 (147.9-451.9) | <0.0001 |
| CX3CL1/Fractalkine | 137.0 (104.1-188.) | 135.0 (73.23-274.8) | ns |
